# Clinical Diagnosis and Perioperative Management of Glioma-Related Epilepsy

**DOI:** 10.3389/fonc.2020.550353

**Published:** 2021-01-14

**Authors:** Gan You, Zhiyi Sha, Tao Jiang

**Affiliations:** ^1^ Department of Neurosurgery, Beijing Tiantan Hospital, Capital Medical University, Beijing, China; ^2^ Department of Neurology, Medical School, University of Minnesota, Minneapolis, MN, United States

**Keywords:** diagnosis, epilepsy, glioma, seizure, surgery, treatment

## Abstract

Gliomas account for more than half of all adult primary brain tumors. Epilepsy is the most common initial clinical presentation in gliomas. Glioma related epilepsy (GRE) is defined as symptomatic epileptic seizures secondary to gliomas, occurring in nearly 50% in high-grade glioma (HGG) patients and up to 90% in patients with low-grade glioma (LGG). Uncontrolled seizures, which have major impact on patients’ quality of life, are caused by multiple factors. Although the anti-seizure medications (ASMs), chemotherapy and radiation therapy are also beneficial for seizure treatment, the overall seizure control for GRE continue to be unsatisfactory. Due to the close relationship between GRE and glioma, surgical resection is often the treatment of choice not only for the tumor treatment, but also for the seizure control. Despite aggressive surgical treatment, there are about 30% of patients continue to have poor seizure control postoperatively. Furthermore, the diagnostic criteria for GRE is not well established. In this review, we propose an algorithm for the diagnosis and perioperative management for GRE.

## Introduction

Epilepsy is among the most common clinical presentations of brain tumors, and seizures often present as the first clinical symptom. Glioma related epilepsy (GRE) is defined as symptomatic epileptic seizures secondary to gliomas. The incidences of epilepsy in patients with gliomas range from 40 to 90%, depending on the tumor types (1–5). The mechanisms of glioma-related epilepsy are thought to be multifactorial and share common pathways with glioma growth processes (6–8). The pathogenesis of GRE involves many factors including tumor histology, tumor location, microenvironment; specific genetic alterations such as isocitrate dehydrogenase 1 (IDH1) gene mutation, O6-methylguanine-DNA methyltransferase (MGMT) promoter methylation; aberrant expression of potassium chloride cotransporter (KCC2), solute carrier family 7 member 11 (SLC7A11), glutamate, etc (8–11). In terms of postoperative seizure control, extent of resection, age, seizure type, seizure duration and Ki-67 expression have been identified as predictors (2, 12).

With the development of new imaging technology, more and more patients with glioma and GRE are diagnosed at an early stage. Nevertheless, the diagnostic criteria for GRE remain controversial, making it difficult to have a treatment guideline for GRE. Therefore, it is very important to have a standard diagnostic criteria for GRE.

Because tumor itself contributes significantly to seizure occurrence, most GRE patients could become seizure-free in the early stage after tumor resection. Gross total resection remains an independent positive predictor for postoperative seizure control (2, 12, 13). However, uncontrolled seizures could occur in 20–40% of patients post resection (2, 4, 11, 14). Recurrent seizures substantially impact quality of life, especially as newer therapies for gliomas have extended life expectancies. As a highly tumor associated disease, GRE is being treated on the second purpose of surgery followed by oncologic tumor control. Surgical treatment of gliomas has the potential to ameliorate seizures when combining with individual and normalized treatment of anti-seizure medications (ASMs) and radiochemotherapy. In this review, we propose an algorithm for the diagnosis and perioperative management for GRE, hoping to provide a better standard for improving seizure control in patients with gliomas.

## Diagnosis of GRE

Unlike patients with idiopathic epilepsy, for patients with GRE, seizure risk is influenced by tumor types. It is important to have comprehensive history-taking and physical examination during the initial visit. Seizure characteristics and relevant clinical information should be documented in details (15). Seizure type should be classified according to the 2017 International League Against Epilepsy (ILAE) guidelines in order to guide management (16). Patients with suspected GRE should receive magnetic resonance imaging (MRI) and electroencephalogram (EEG) examinations. The diagnosis of GRE should be based on both the clinical information and imaging findings.

First of all, for the diagnosis of glioma, MRI with T2-weighted and contrast-enhanced T1-weighted imaging are essential. The other conventional sequences, such as diffusion-weighted imaging (DWI), perfusion-weighted imaging (PWI) and fluid-attenuated inversion recovery imaging (FLAIR) should also be included. In addition, computed tomography (CT), magnetic resonance spectroscopy (MRS) and positron emission tomography (PET) are also helpful for differential diagnosis. Pathological evaluation is the gold standard for the diagnosis of glioma. For the 2016 World Health Organization (WHO) classification (17), molecular pathological examination should be performed.

Secondly, for the diagnosis of epilepsy, a video EEG for more than 2 hours is recommended for patients with definite or possible seizure history (15). Ictal EEG is helpful for the differential diagnosis to rule out non-epileptic attacks in patients without typical clinical seizures or interictal epileptiform discharges (15, 18). PET, single-photon emission CT (SPECT), and magnetoencephalography (MEG) can also be used to localize the epileptogenic zone (19, 20).

**Figure 1:**
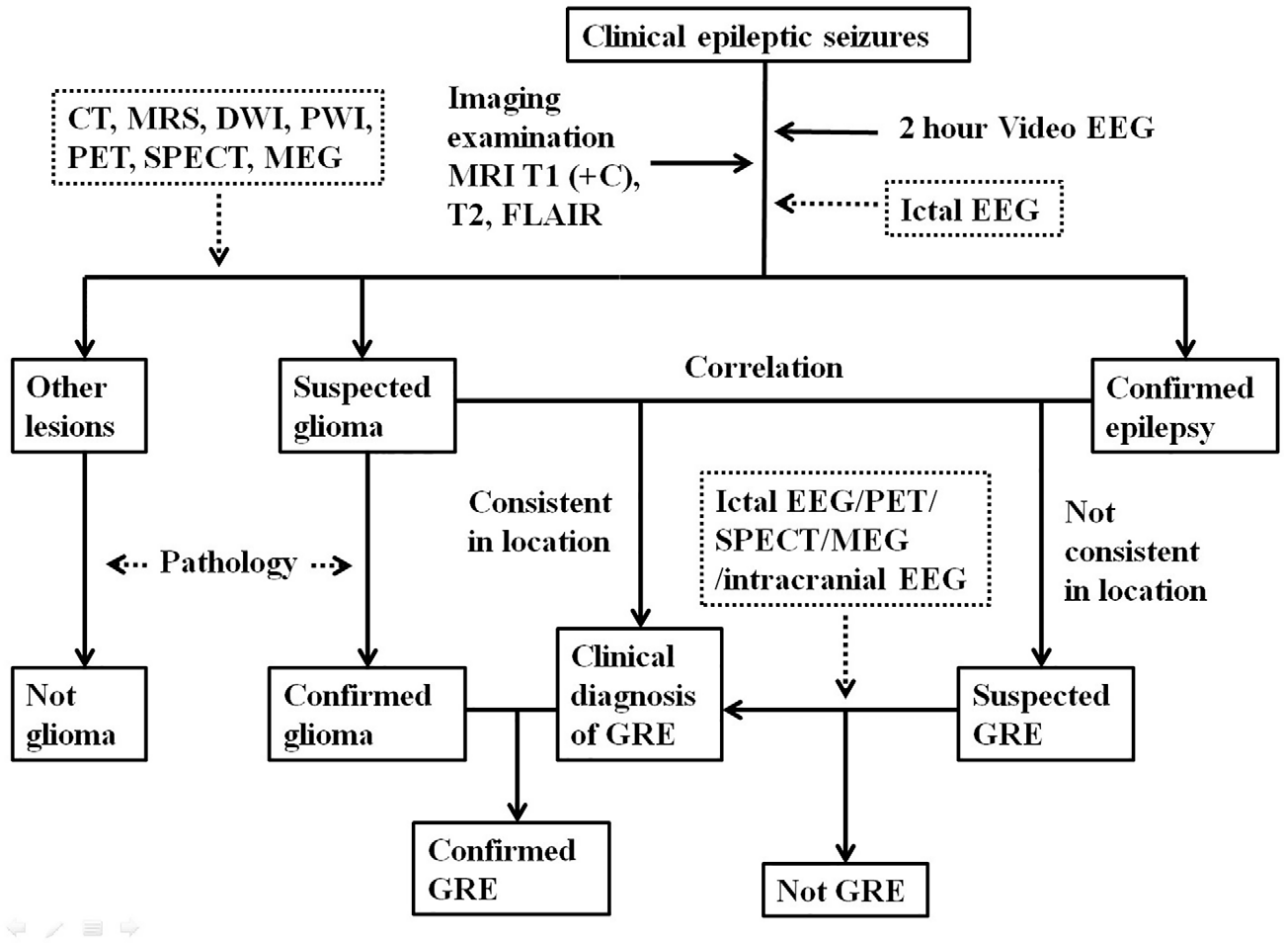
The diagnostic algorithm of glioma-related epilepsy.

To make the diagnosis of GRE, a clear correlation between glioma and epilepsy should be established. Although intracranial video EEG is rarely performed for patients with GRE due to significant cost and risks, this is a useful diagnostic tool for cases that show no clear causal relationship between the tumor and seizures. Intracranial video EEG monitoring is recommended for epilepsy patients whose epileptogenic zones are thought to be unrelated to the brain tumor (15). A preoperative diagnosis of GRE is generally considered a clinical diagnosis which mainly depends on clinical signs, EEG, imaging studies. A diagnosis of GRE can only be confirmed after pathological validation (**Figure 1**).

## Preoperative ASMS Application for GRE

It is generally recommended to start ASMs medication when epilepsy is confirmed preoperatively because of great risk of seizure recurrence (21). The selection of ASMs should follow an individualized treatment plan based on age, sex, comorbidity, and concurrent medication therapy. One of the principles for the initial ASMs use in GRE patients is monotherapy with adequate drug dosage and duration. Given the fact that concomitant use of steroids and chemotherapy is common for postoperative treatment of glioma, ASMs with potential drug interactions as well as hepatic enzyme induction effects should be avoided (22, 23). At present time, evidence based medicine had suggested that the drug of choice for AED monotherapy for GRE are levetiracetam (LEV) and valproic acid (VPA) (24–26).

Recent studies on GRE treatment using LEV have suggested good efficacy and favorable safety profile (27, 28). With the advantages of improving the cognitive functioning and enhancing the chemotherapeutic efficacy of Temozolomide (TMZ), LEV is widely used for treating GRE as either monotherapy or add-on therapy (28, 29). VPA is a broad spectrum, well-tolerated AED that is widely used in epilepsy with brain tumors. As a histone deacetylase inhibitor, VPA may also inhibit glioma-genesis and prolong survival in patients with glioblastoma (GBM) (7, 30). If the first monotherapy AED has insufficient seizure control, switching to a different AED may be considered. Recently, there is a trend for polytherapy with combination of VPA with LEV if monotherapy with either one is ineffective (7, 25).

If neither LEV nor VPA or their combination is ineffective for seizure control, lacosamide (LCM), perampanel (PER), lamotrigine (LTG), pregabalin (PGB), zonisamide (ZON), and Brivaracetam (BRV) may be considered as add-on ASMs in GRE. LCM has a good efficacy and fewer side effects in GRE patients compared to other ASMs (31). LCM was also shown to have antineoplastic effect on glioma cells *in vitro* (32). PER, which functions as a noncompetitive AMPA receptor antagonist (33), was shown to inhibit GBM cell growth and glutamate release *in vitro* (34). Seizure-freedom periods could be achieved in drug-resistant epilepsy in patients with GRE when PER was used (35, 36). Because of their good tolerability and their synergistic activities with VPA, LTG, PGB, and ZON could also be considered as add-on agents for GRE (24, 29, 37). Brivaracetam is also a promising antiepileptic drug in this group of patients (38). We summarized the main characteristics of the antiepileptic drugs mentioned above in **Table 1** (21, 24, 28, 39).

**Table 1:** Main Characteristics of ASMs for GRE patients.

ASMs	Recommended dosage (mg/day)	Intravenous formulation	Main mechanism of action	Common adverse events	Drug interactions with other ASMs*
VPA	500–2,500	Yes	GABA-receptor agonist, Na^+^-channel blocker, Glutaminergic inhibitor	Hepatotoxicity, thrombo- and neutropenia, tremor, weight gain, hair loss, ovarian cystic, syndrome	LTG
LEV	1,000–3,000	Yes	Binding to synaptic vesicle protein 2 (SV2A)	Somnolence, asthenia, irritability, psychosis	LTG
LCM	200–400	Yes	Slow Na^+^-channel blocker	Dizziness, headache, nausea, diplopia, blurred vision, cognitive, dysfunction, skin reactions	
PER	2–12	No	AMPA receptor antagonist	Somnolence, dizziness	LTG
LTG	200–600	No	Na^+^-channel blocker	Rash, SJS/TEN, DRESS, headache, ataxia	VPA, LEV, PER
PGB	150–600	No	Ca^+^-channel blocker	Somnolence, dizziness, ataxia	
ZON	200–600	No	Na^+^-and Ca^+^-channel blocker	Somnolence, ataxia, dizziness, renal calculi	
BRV	200–400	Yes	Binding to synaptic vesicle protein 2 (SV2A)	Somnolence, dizziness	

*Limited to the ASMs mentioned in this table.

For patients without preoperative seizures, most studies and clinical guidelines advise against prophylactic ASMs use before surgery in patients with glioma (40, 41) unless they have the following risk factors: younger age, temporal lobe gliomas and cortex involving gliomas (2, 4, 42, 43).

## Surgical Management of GRE

The goal of surgery in patients with GRE is not only tumor removal, but also seizure control, especially for LGG patients who usually have longer survival. Gross total resection is a known predictor of seizure freedom in glioma (2, 44). For gliomas with deep location or involving eloquent cortex, advanced intraoperative technologies, such as neuronavigation, awake surgery and “engraving surgery”, are recommended to maximize resection while preserving function. Electrocorticography (ECoG) in the intraoperative setting can help to identify epileptogenic zone to guide resection. ECoG has been shown of great value for LGG patients with preoperative refractory GRE (45). In addition, supratotal resection in noneloquent area can be helpful to optimize seizure control (6, 46).

Intraoperative direct electrical stimulation for functional cortical or subcortical mapping may induce seizures during awake craniotomy for gliomas. The incidence is approximately 8% (47). Patients with the following risk factors are more likely to experience intraoperative seizures: younger age, supplementary motor area involvement, preoperative seizure history, and IDH1 mutation (43, 47, 48). Prophylactic intravenous infusion of LEV or VPA half an hour before surgery can be used for these high-risk patients. The surgeons must stop stimulation immediately and irrigate the cortex with ice-cold Ringer’s solution or saline as soon as possible once intraoperative seizures occur (49). In case of status epilepticus, benzodiazepines or anesthesia enhancement should be initiated in a timely manner. It is worth mentioning that intraoperative electromyographic monitoring and ECoG could usually detect potential seizure onset early (43, 45).

## Treatment of Early Postoperative Seizures

Early postoperative seizures (EPS) occur within the first week of surgery. Although clinical evidences have suggested no benefit for prophylactic ASMs during the perioperative period for GRE patients (40, 50), AED prophylaxis are still used routinely in clinical practice (51). One of the goals is to minimize the risk of EPS. Given the increased risk of EPS due to surgery, ASMs may be considered for 1 week after surgical resection whether or not patients had history of GRE. To evaluate the extent of tumor resection accurately, postoperative MRI with contrast-enhancement should be performed within 24–72 h after surgery.

In the case of EPS, intravenous or intramuscular injection of short acting ASMs should be administered as soon as possible to avoid status epilepticus, followed by performing electrocardiography and blood tests to exclude conditions caused by cardiac dysfunction, hypoglycemia, or electrolytes disturbances. Subsequently, CT or MRI must be performed to exclude intracranial hemorrhage, infarction and pneumocephalus. If the patient did not receive prophylactic ASMs perioperatively, AED monotherapy should be used (52). The blood concentrations of ASMs should be monitored regularly when more than two seizure episodes occur and add-on treatment with a second AED must be considered when seizures are not controlled with a single agent (43). A 2 h EEG monitoring is recommended to evaluate the interictal activities and to guide subsequent medication use (15).

## Long-Term Postoperative Management of GRE

The majority of GRE can be managed effectively by either monotherapy or with combination of ASMs. The choice of ASMs should be individualized based on patients’ responses and comorbidities. Recent evidences have shown that antitumor treatment with radiotherapy and chemotherapy may lead to improved seizure control (6, 53). Therefore, the potential interaction with chemotherapy agents should be considered when ASMs is selected. Given the fact that there are few known harmful drug interactions of LEV and VPA with chemotherapeutic agents (22, 23), LEV and VPA are recommended for monotherapy in patients with GRE after surgery to improve seizure control and quality of life (25, 26, 29, 54).

Some patients with glioma and GRE may achieve long-term seizure freedom after gross total resection and adjunct antitumor therapy, raising doubts about the necessity to continue ASMs. The withdrawal of ASMs remains a complex and controversial issue for glioma patients, as seizure risk is highly influenced by tumor types, stages and location, and tumor treatment. It remains very difficult to predict the precise risk of seizure recurrence in GRE patients. In general, it is recommended to avoid AED withdrawal in patients with high risk of seizure recurrence (55). This relapse risk depends on various factors including, but not limited to, patient age, seizure types, tumor location and grade, the extent of a surgical resection, tumor genetic features and types of antitumor treatment (2, 4, 34, 55). A recent study has suggested that AED withdrawal should only be considered in patients with a low risk of tumor progression, even the patients had experienced long-term seizure freedom (56). However, long-time use of AED may lead to significant side effects and should be only given to carefully selected patients. At present time, guidelines regarding the timing of ASMs withdrawal are urgently needed for GRE patients. Based on current evidence (2, 4, 42–44, 55–57), we propose the following recommendations in regarding the timing of ASMs withdrawal for patients with GRE (**Table 2**):

1. For patients without preoperative seizures and EPS, prophylactic ASMs should be withdrawal 2 weeks after surgery.
2. For patients with preoperative low-risk seizures (generalized seizure type, short-term seizure duration and drug controlled seizures) and without EPS, ASMs may be tapered off after 3 months postoperatively.
3. For patients with preoperative high-risk seizures (focal seizure type, long-term seizure duration and uncontrolled seizures), EPS or repeated postoperative seizures, ASMs withdrawal should be considered after a minimum of 1 year of seizure freedom.
4. For patients with incomplete tumor resection, intraoperative distant epileptic discharges on ECoG or epileptic discharges on EEG at follow-up, AED should be withdrawn no sooner than 2 years seizures freedom after the surgery.
5. For patients with the following tumor/surgery related risk factors, the postoperative duration of ASMs should be prolonged accordingly: (a) severe cortical injury during tumor resection; (b) an extended surgical procedure (cortex exposed for over four hours); (c) postoperative brain edema or cortex hemorrhage/infarction; (d) re-operation due to emergency; (e) gliomas with an oligodendroglial component; (f) recurrent gliomas.
6. For all patients with GBM or anaplastic glioma without complete tumor resection, AED withdrawal is not recommended.

**Table 2:** Recommendation of Timing of ASMs Withdrawal for Patients with GRE.

Timing of ASMs withdraw	Patients with preoperative seizures			Patients without preoperative seizures			Patients with incomplete tumor resection or with epileptic discharges		Patients with tumor/surgery-related risk factors^§^
	High risk group^#^	Low risk group		EPS	No EPS		LGGs	HGGs	
		EPS	No EPS						
2 weeks after surgery						•			
3 months after surgery		•^*^	•	•^*^					•
After 1 year of seizure freedom	•	•		•					•
After 2 year of seizure freedom	•						•		•
Lifelong Medication								•	

^#^Risk factors for poor postoperative seizure control.

Focal seizure type. Long seizure history (> 1 year). Preoperative uncontrolled seizure. Preoperative multiple ASMs. ≥1 EPS. Epileptic discharges on EEG at follow-up.

^*^Only 1 episode of EPS.

^§^Tumor/surgery-related risk factors.

Severe cortical injury during tumor resection. An extended surgical procedure (cortex exposed for over four hours). Intraoperative distant epileptic discharges on ECoG. Postoperative brain edema or cortex hemorrhage/infarction. Re-operation due to emergency. Gliomas with an oligodendroglial component. Recurrent gliomas.

Previous studies have shown that seizure recurrence following a prolonged seizure-free period could be suggestive of tumor progression, which would require prompt repeat imaging and re-evaluation of ASMs (11, 52). If patients suffer from postoperative seizures without evidence of tumor recurrence, adjustment of ASMs should be considered after adequate AED blood levels are confirmed. In cases of drug resistant GRE, epilepsy surgery or radiotherapy could be considered when quality of life is significantly impacted due to frequent seizures (15, 43, 53). Other factors, such as side effects from ASMs, financial and psychosocial aspects should also be considered when managing drug resistant GRE.

## Conclusions

Diagnosis of GRE should be based on the coexistence and causal relationship of glioma and epilepsy. Seizure prognosis should be considered as important factor in addition to survival in managing GRE, especially for patients with LGG. Gross total resection of tumor is the most effective therapies for GRE. Intraoperative ECoG and postoperative EEG are useful for patients with perioperative refractory GRE. Standardized ASMs application and appropriate antitumor treatment are highly recommended for the management of GRE. Timing of postoperative ASMs withdrawal should be individualized based on perioperative risk factors.

## Future Prospects

Accurate diagnosis of GRE is important from stand point of view of both neuro-oncology and epileptology. Accurate localization seizure onset zone and optimal tumor resection for gliomas may be achieved by incorporating tools used in epilepsy surgery and contemporary neuroimaging technologies. Large well-designed randomized controlled trials are needed to determine the usage of prophylaxis ASMs perioperatively in GRE. Possible survival benefits from ASMs therapy should be further investigated and confirmed. More genetic research is needed to further explore the underlying pathogenesis, and eventually to provide effective treatment for GRE.

## Author Contributions

GY wrote the manuscript. ZS and TJ revised the manuscript. All authors contributed to the article and approved the submitted version.

## Funding

This work was supported by the National Natural Science Foundation of China (No.81871013), Beijing Municipal Education Commission Science and Technology Plan General Project (No.1192050172), and Beijing Tiantan Hospital Young Scientist Program (YSP201705).

## Conflict of Interest

The authors declare that the research was conducted in the absence of any commercial or financial relationships that could be construed as a potential conflict of interest.

## Abbreviations

WHO, World Health Organization; ILAE, International League Against Epilepsy; LGG, low-grade glioma; HGG, high-grade glioma; GBM, glioblastoma; GRE, glioma related epilepsy; ASMs, anti-seizure medications; EEG, electroencephalogram; ECoG, electrocorticography; EPS, early postoperative seizures; IDH1, isocitrate dehydrogenase 1; MGMT, O6-methylguanine-DNA methyltransferase; KCC2, potassium chloride cotransporter; SLC7A11, solute carrier family 7 member 11; MRI, magnetic resonance imaging; DWI, diffusion-weighted imaging; PWI, perfusion-weighted imaging; FLAIR, fluid-attenuated inversion recovery imaging; MRS, magnetic resonance spectroscopy; CT, computed tomography; PET, positron emission tomography; SPECT, single-photon emission CT; MEG, magnetoencephalography; TMZ, temozolomide; LEV, levetiracetam; VPA, valproic acid; LCM, lacosamide; PER, perampanel; LTG, lamotrigine; PGB, pregabalin; ZON, zonisamide; BRV, brivaracetam.
